# The possibilities of day surgery system development within the health policy in Slovakia

**DOI:** 10.1186/s13561-014-0035-1

**Published:** 2014-12-28

**Authors:** Vincent Šoltés, Beáta Gavurová

**Affiliations:** Faculty of Economics, Technical University of Kosice, Nemcovej 32, Kosice, 040 01 Slovakia

**Keywords:** Health policy, Health financing policy, System of one-day surgery, Efficiency of health care, Correspondence analysis, Methodological problems of reporting

## Abstract

**Background:**

In the day surgery system are intertwined elements of state health policy, health care payers’ interests, employers of health care system, as well as the interests and wishes of patients. A problem in the health policy is to find a way to regulate ambulatory and short-term surgical procedures, which are hardly distinguishable, and still fulfil the requirements of transparent financing, quality and security. The objective of this paper is to highlight the reasons for the long-term stagnation in Slovakia day surgery and the possibilities of eliminating the structural drivers causing this negative phenomenon.

**Methods:**

Due to the nature of the analyzed data and desired outcomes, we selected application of correspondence analysis. Results of correspondence analysis provide valuable information necessary for the projection of specialization of one day surgery clinics for that type of procedure, as well as for the support of the new clinics creation (also with the potential state support), the pricing policy, systemic reduction of beds what is connected with reduction of underutilized departments in hospitals, in order to optimize management processes in the healthcare system.

**Results:**

Contribution reveals negative aspects which causing a low level of day surgery in Slovakia. Moreover, it reveals the approaches of the different subjects of day surgery. Presented options for setting optimal strategy supporting its development are based on the results of the analysis. Correspondence analysis provided valuable information of present structure of the day surgery system. The determined similarity of the regions and association of specialized fields indicate specific settings of the day surgery system and its components that are inevitable to analyze in the subsequent analytical process.

**Conclusions:**

Results of the analysis are very important in order to set up the system measures in the process of its further development, which should be part of the strategic plan of each health system. On conceptual and methodological issues related with reporting of day surgery performances are highlighting international organizations such as the OECD,WHO.

**JEL classification:**

I13, I18, H51

## Background

Healthcare has limited sources and consequently, the total demand of healthcare may not be met. Even the basis of economic scarcity law results into a belief that production of healthcare inevitably leads to discrimination of some needs due to this fact. Price discrimination represents a natural way of solving a problem based on source limitation and it is typical for our health system, as well [1]. The present procedures in treatment of some illnesses types are very costly and in their payment, there will not be left any resources for treatment of other patients. Therefore, a significant role plays a regulation of health systems with characteristic qualities of political solution of some economic issues and central planning from state’s side [2-4]. Since 2002, the Slovak health system has been evident by soundly increasing indebtedness, whose primary reason is ineffective disposal of public sources in a system together with soft budget criteria of state allowance organizations. In this period of time, public especially deals with an issue of implementation and use of day surgery (DS) that is defined as one of very effective ways of providing healthcare, which also represents a benefit for many patients as well as health insurance companies (HIC) [5,6]. The process of reducing the bed facilities that was also a part of criticized restructuring process of a government due to non-critical treating of foreign models has begun since 2007. However, DS did not perform its role of a full-valued indicator in the process of healthcare optimization and it was usually realized in the departments that provide a traditional hospitalized care. At that times, a rise of DS centres in the world occurred, while numerous research studies declared notable differences in a range of using DS as well as in a reached level of efficiency [7,8]. The medical reasons do not represent the main reasons, but the health policy and a searching for possibilities in its system, as well as a regulation of those procedures in the state, refunding and subsequently, provision of their necessary quality and safety [9]. At present, the results of the own research activities prove that the DS system in Slovakia is functional, but its individual components are not correctly set, which leads to DS stagnation for many years [10,11]. Its progress requires effective regulation of health system that especially makes provision for health needs of inhabitants [12-14]. Their finding is very difficult especially from methodological aspect (it is given by a range, structure, representativeness and credibility of available data). Slovakia lacked any research studies and analyses that would deal with DS issues till 2013. Therefore, the process of bed reduction was followed by a separate solution of day surgery, as well as classical hospitalized care [15]. The partial aims that lead to reaching the main aim are represented by an evaluation of the significance of these analyses in the Slovak health system, meeting all the requirements of data-base high quality, the connections to the issue of reporting the proclaimed international institutions, such as OECD, WHO and Eurostat, which are related to these analyses. An adequate attention to these issues in a national range will enable the Slovak health system to prepare a suitable platform for implementation of the DS system developing components, benchmarking, and also to provide its development that will be comparable with other countries.

### Reporting issues in national and international indexes

**Figure 1:**
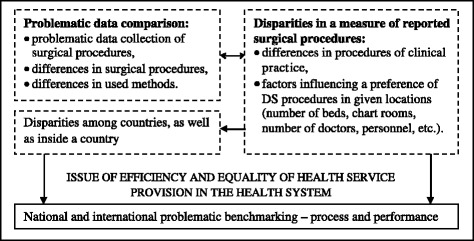
**Reporting process of the surgical procedures – determinants and their connections.**

The international organizations OECD and Eurostat have been gathering data of surgical procedures as a part of a total collection of data that provide an image of healthcare for many years. Collection of these data is highly difficult and some factors that cause significant disparities in a measure of reported surgical procedures in the individual European countries (Figure 1) make it even more difficult.

The centre of these issues may be visible in the process aspect of reporting of the realized health processes (or procedures). It is an important determinant that influences a rate of reported surgical procedures and generates the primary issues in a data area. These are aggregated into three main constitutive levels that will be closely introduced.

Firstly, it deals with issues that are connected with using *significantly differentiated methods in gathering surgical procedures data*. OECD and Eurostat differed in a process of data collection by segmentation of procedures into “bed cases” and “daily cases”, while the definition and data gathering of daily cases were unclear and inconsistent [16]. The second level is *heterogeneity of methods that are used in a correct reporting of realized* procedures, which is conditioned by the first constitutive level (while not emphasizing a place of realization as in the first case, but a type of procedure). Three types of reporting methods were found out by analysis: those that emerge from a number of all procedures that are given in a record of hospital discharge, those that only emerge from main procedure (they do not respect secondary procedure) and those that emerge from a number of patients with applied procedure during their stay in a hospital. The given reasons lead to the point, when total number of realized procedures may be even higher than the reported ones on the basis of various instructions, which predominantly depends on a detailed division of some national classified systems. Also the OECD, WHO and Eurostat institutions have different approach to the process of data collection. OECD and Eurostat had been realizing the data collection of surgical procedures by means of two files till 2010: file of aggregated data with reported number of all procedures, as well as a file with a detailed data that are related to chosen procedures.

WHO-Europe focused this process of data collection on total results that were related to the file of aggregated data (number of all types of surgical procedures in a group of bed, as well as daily cases), as well as structured data on the basis of a classification of surgical procedures (high number, high costs, etc. belong to classified criteria that also respect a division into bed and daily cases). Trajectory of primary macro-economic issues that are related to reporting the data for international comparisons are completed with third constitutive level – this is conditioned by variability of components that form the definitions of procedures, which are used in a process of data collection of surgical procedures. The differences in definitions cause significant issues in announcing the consistent and comparable data. The definition according to Eurostat has more general and wider character as the definition given by OECD and WHO-Europe. The reason is an implementation of surgical and also other procedures according to the definition. This fact was significantly consequent in relation to reporting a higher number of procedures submitted for Eurostat, in comparison to reported data for OECD, or WHO-Europe.

Only 4 countries out of 26 analyzed countries do not have any reported data of surgical procedures: France, Iceland, Norway and Slovakia, according to the lately available statistics. Regarding the given issues in macro-standard, the data collected by international institutions are considered as inappropriate for any type of benchmarking as a consequence of their insufficient reliability and validity^a^. We suppose that these reasons also supported a decision of international organizations to stop the data collection of total number of surgical procedures till the international classification of procedures will not be agreed and implemented. This classification guarantees a higher consistency of reported data among individual countries. It is also inevitable to adjust and correct a methodology inside each country, to provide a transparent data collection, required interoperability of IT systems and especially IT discipline of healthcare providers (reporting micro-level). In Slovakia, the data of surgical procedures are collected by means of a standardized report (NHIC) since 2009, while we criticize its inappropriate and insufficient structure. Our analyses [15] supported a proposal of its change by presently acceptable and competent institutions in Slovakia. However, the issues in this area are deeply systemic and they are related to politics, financing of the DS procedures (non-transparent system of refunds of the DS procedures by health insurance companies), conditioned by unfair behaviour of some healthcare providers (HCP) in the report process and by ignoring the significance of reporting, which forms a negative synergic effect in the whole process. It is inevitable to understand a large strategic significance of a position and tasks of national register in the health policy of the state, as well as in an international context.

### Day surgery and its significance in the system of healthcare efficiency

DS solves morbid conditions that may not be solved by a single visit of a doctor, but they also do not require several-day bed hospitalization [17-20]. The fundamental principle is based on the fact that in DS procedures are not required more than 24 hours the post-operative care. It is advantageous to patients and HIC, and it is also supported by governmental program of Ministry of Health of the Slovak Republic within a system of bed reduction in the hospitals and transfer of health care to more effective ambulance care. It is realized on the basis of Regulation of Ministry of Health of the Slovak Republic^b^. Positive or negative opinion of its use in Slovakia depends on a person who judges it. It is differently perceived by the HIC which struggle to use available financial means effectively, and differently perceived by permanently indebting hospitals that occur in this situation due to lower payments for DS procedures against payments for terminated hospitalization. The main cause is probably wrongly set and economically demotivated system that causes a significant leeway in DS behind all-European average (7-10% of all surgical procedures).

DS development in the individual countries is significantly differentiated and influenced by changes of their health systems. For instance, the USA and Canada present over 65-70% of day surgery procedures out of the total operations (e.g. [21,22]), 43% out of all operational procedures in Sweden [23], 51% of planned operations in the UK and 61% of planned operations in Norway. It is caused by diametrically different processes of the DS development that also have effectively developed and interconnected support social programs [24-26]. Despite the legislative support of the Ministry of Health of the Slovak Republic, the DS development process in Slovakia has not fully-developed so far. The best medical fields in the DS functioning are Ophthalmology, Otorhinolaryngology and Gynecology. New conditions in the DS provision have been effective since 2009. The Official Gazette contained 450 DS procedures of surgically medical fields, out of which approximately 130 procedures were related to child patients. Many child specialists – surgeons supported this decision of the Ministry of Health of the Slovak Republic, in spite of the fact that the conditions for DS provision for children are stricter than for adults (legislatively and medically). The data of the DS procedures are collected through the whole area in all medical workplaces in the regions on the basis of standardized report since 2009, and they are legislatively adjusted by the Ministry of Health of the Slovak Republic.

## Methods

The selection of CA is based on the nature of the data which performed structured standardized data available on the basis of contractual cooperation with NHIC. Secondly, applications of the opportunities that provide CA in connection with our analysis of DS system and were crucial for our analysis. The reason for the selection of these examined sample is contingent, firstly, by the structure of the disposable data and secondly, by the nature and conditions CA. One of the prerequisites of the correspondence analysis is the completeness of characters, thus variables. In this case, we have a very large number of combinations of two category variables of zero. For example, in the Bratislava region, except surgery, four other departments perform zero number of performances, which is very extreme. It is also very difficult to implement correspondence analysis using data that are available for hospitalized elderly. This fact also relates to the situation that juniors do not show such morbidity and thus the need for any subsequent hospitalization than people over 18 years. We realized a depth analysis of structure of DS procedures of child patient in order to reach the main aim (according to the Regulation of the Ministry of Health of the Slovak Republic, it is a category of children to 18 years old). We wanted to find the similarities of procedures’ structure in the individual regions and to reveal the connections among them. Its positive progress will not be possible in the future without any effective measurements, analyses and implantations of their procedures into the DS system and its subsequent transformation into a strategic intention of the Slovak health system. The required data of DS procedures were available on the basis of contractual cooperation between the NHIC and the Ministry of Health of the Slovak Republic, while the subject of our analysis involves data for 2012.

### Methodology of correspondence analysis

We have fulfilled our primary aim by choosing the application of correspondence analysis (CA) regarding the character of researched issue and a structure of obtained data. This application is very rarely used in Slovakia. CA was developed as a method for graphically displaying rows and columns of two-dimensional (later known as multi-dimensional) cross table (pivot tables). The basis of CA lies in data arrangement of relative frequencies – shares into main components. As it represents the frequency of categorical (qualitative) data, it has its specifications.

The graphs of horizontal, column or total shares that mostly occur in the first two main components, where geometrically close (remote) points also represent numerically close (different) shares are the results of CA [27]. In this way, it helps the interpretation of found and significant connections of the analyzed facts.

We focused on the way of its calculation in applying the CA as Hintze [28,29] presents in his works:

1. Entering data matrix ***K*** with the *n* (rows) and *m* (columns). Note that the elements of ***K*** must be non-negative and that none of the row or column totals is zero (we cannot have zero rows/columns).

2. Compute the proportion matrix ***P*** by dividing the elements of ***K*** by the total of all numbers in ***K***. Mathematically, we write:

1 $$ \mathbf{P}=\left\{{p}_{ij}\right\}=\left\{{k}_{ij}/k\right\} $$

3. Compute the totals of the rows and the columns of **P**, putting the results in the vectors **r** and **c**. By using the standard matrix notation, we write:

2 $$ \mathbf{r}=\mathbf{P}\mathbf{1} $$

3 $$ \mathbf{c}=\mathbf{P}\hbox{'}\mathbf{1} $$

where **1** is an appropriately dimensioned unit vector.

4. Change the square roots of the vectors **r** and **c** into diagonal matrices and take the inverse of the resulting square matrices.

4 $$ {\mathbf{D}}_r={\left[ diag\left(\mathbf{r}\right)\right]}^{-1/2} $$

5 $$ {\mathbf{D}}_c={\left[ diag\left(\mathbf{c}\right)\right]}^{-1/2} $$

5. Compute the scaled matrix A:

6 $$ \mathbf{A}={\mathbf{D}}_r\mathbf{P}{\mathbf{D}}_c $$

6. Decomposition of matrix **A** into singular values [30,31] – acronym SVD (Singular Value Decomposition). In the publications of linear algebra, it sometimes occurs as LDU decomposition – as a generalization of Cholesky LU decomposition [32]:

7 $$ \left\langle \mathbf{B},\mathbf{W},\mathbf{C}\right\rangle =SVD\left(\mathbf{A}\right) $$

7. Compute the coordinate matrices **F** and **G** as follows:

8 $$ \mathbf{F}={\mathbf{D}}_r\mathbf{B}\mathbf{W} $$

9 $$ \mathbf{G}={\mathbf{D}}_c\mathbf{C}{\mathbf{W}}^{\mathbf{\prime}} $$

8. Compute the eigen values, V:

10 $$ \mathbf{V}=\mathbf{W}{\mathbf{W}}^{\mathbf{\prime}} $$

9. Compute the row distances, *d*_*i*_ and the column distances *d*_*j*_:

11 $$ {d}_i={\displaystyle \sum_j\left(\frac{1}{p_{.j}}\right)}{\left(\frac{p_{ij}}{p_{i.}}-{p}_{.j}\right)}^2 $$

12 $$ {d}_j={\displaystyle \sum_i\left(\frac{1}{p_{i.}}\right)}{\left(\frac{p_{ij}}{p_{.j}}-{p}_{i.}\right)}^2 $$

10. Note that the weights, *w*_*i*_ and *w*_*j*_ come from vectors *r* and *c* (these were formed in step 3).

13 $$ {w}_i=\kern0.5em \left\{{r}_i\right\} $$

14 $$ {w}_j=\kern0.5em \left\{{c}_j\right\} $$

11. Designation and calculation of the various parameters:

Weight - *w*_*i*_, row factor - *f*_*ij*_, column factor - *g*_*ij*_.

Inertia:

15 $$ \frac{w_i{d}_i^2}{{\displaystyle \sum_k{w}_k^2{d}_k^2}} $$

Distance:

16 $$ {d}_i^2 $$

Row COR:

17 $$ \frac{f_{ij}^2}{d_i^2} $$

Column COR:

18 $$ \frac{g_{ij}^2}{d_j^2} $$

Row CTR:

19 $$ \frac{w_i{f}_{ij}^2}{v_i} $$

Column CTR:

20 $$ \frac{w_j{g}_{ij}^2}{v_j} $$

Angle:

21 $$ ArcCos\left(\sqrt{CO{R}_{ij}}\right) $$

Decomposition of matrix into singular values is a generalization of decomposition into own values. Its basis is a decomposition of a given matrix into composition of three matrices, so-called left singular = left bottom matrix (it has non-zero members only below its main diagonal), diagonal (it contains positive diagonal elements – singular values) and right singular = right upper matrix (it contains non-zero members only above its main diagonal). In case of symmetric entry matrix, there is a singular decomposition same as the spectral decomposition. Then, the right and left singular matrix is equal to their own vectors and singular values are own values. The primary reason of using the singular values in matrix decomposition is their robustness to defects in the matrix elements, which is not a case of own values in non-symmetric matrices [31].

The mass – weight is a share of row total to overall total of a table.

The inertia is the whole table is a function of the Chi-square statistics χ^2^. If

22 $$ {\chi}^2={\displaystyle \sum_{all\kern0.5em i,j}\frac{{\left({O}_{ij}-{E}_{ij}\right)}^2}{E_{ij}}} $$

where *O*_*ij*_ is the count of row *i* and column *j* of the table, *E*_*ij*_ is the value expected under the assumption of row-by-column independence, and *N* is a total table count, then the total inertia of the table is given by:

23 $$ Total\kern0.5em  Inertia=\frac{\chi^2}{N} $$

The inertia value reported is the proportion of the total inertia that is due to this profile. Another way to interpret the inertia is that it is the weighted average of the Chi-square distances between the row profiles and their average profile.

Factor is the coordinate of the profile along this axis. Correlation (COR) is a correlation between this profile and the inertia of this axis. It is a share of profile dispersion that is explained by axis. This is the proportion of variance in the axis accounted for by this profile. Contribution (CTR) represents an opposite condition, i.e. it explains of this profile to the inertia of this axis. This is the proportion of variance in the axis accounted for by this profile.

### Data – bases

The base of the DS structure analysis is formed by data that were provided by NHIC – the Annual Report J (the Ministry of Health of the Slovak Republic) of day surgery in 2012 with given numbers of patients, which realized a procedure of a given type according to a code of the DS procedures’ code list. This was formed on the basis of a list of procedures published in the Official Gazette of the Ministry of Health of the Slovak Republic on the first of March, 2006 (Regulation the Ministry of Health of the Slovak Republic). The basic structure of the report is illustrated in Table 1.

**Table 1:** **Annual report J(MOH SR) 1-01** ^a^

**Type of day surgery procedure**	**Day surgery procedure code**	**Number of patients**			
		**Operated**		**From that hospitalized after surgery**	
		**Age category juniors** ^**b**^	**Age category adults**	**Age category juniors** ^**b**^	**Age category adults**
		**0 - 18**	**19+**	**0 - 18**	**19+**
Procedure	a	1	2	3	4

^a^Annual Report J(MOH SR) 1–01 and the Official Gazette of the Ministry of Health of SR from 1.3.2006, quota 9–16, part 23 – Regulation of MOH SR of DS procedures.

^b^As NHIC does not have specifically structured categories of children and adolescents, we will use the appellation junior for all of age 0–18.

Source: NHIC.

The given data represent an aggregation of occurrence of individual surgical procedures in 2012 in Slovakia. The Official Gazette of the Ministry of Health of the Slovak Republic presents seven specialized fields of DS procedures: Surgery, Orthopaedics, Surgical Emergency and Plastic Surgery (hereinafter Surgery), Gynecology and Obstetrics, Ophthalmology, Otorhinolaryngology, Urology, Dental Surgery and Gastroenterological Surgery and Gastroenterology. The DS procedures for the last two fields are presented in a minimum extent and thus, we did not involve them into next analyses.

## Results

Firstly, we had formed a correspondence table of procedure numbers of non-hospitalized juniors (a patient leaves a health facility till 24 hours) on the basis of report data (Table 1) according to fields and regions (Table 2). We consider the DS procedures of hospitalized juniors in connection to the definition of DS with a given time of healthcare that is longer than 24 hours as risky and they represent an additional financial cost ratio of given procedures. The reason is a payment system of DS procedure expenses that has a form of a lump sum payment without any regard to other needs of a hospitalized patient. We abstracted the hospitalized juniors from analysis procedures due to problematic evaluation of primary reasons to hospitalize a patient as a consequence of necessary data absence. We suppose that in some of the cases (for instance in young age, type of procedure) there may be recommended a stay in a hospital through the night by a doctor after the DS procedure as a consequence of monitoring and prevention of possible difficulties.

**Table 2:** **Correspondence table of the DS procedure number of non- hospitalized juniors**

**Region**	**Field**					
	**Surgery**	**Gyn**	**Ophthal**	**ORL**	**Urology**	**Active margin**
BL	453	0	3	615	19	1090
TA	171	6	0	359	149	685
TC	192	15	4	536	41	788
NI	104	8	8	726	170	1016
ZI	550	17	54	1491	136	2248
BC	467	71	8	148	6	700
PV	253	30	42	1883	38	2246
KI	269	93	49	1532	786	2729
Active margin	2459	240	168	7290	1345	11502

Abbreviations to the table:

Specialized Field:

SURG: Surgery, Orthopedics, Department of Trauma and Plastic Surgery, GYN: Gynaecology and Obstetrics, OPHT: Ophthalmology, ORL: Otorhinolaryngology, UROL: Urology.

Region:

BC: Banská Bystrica, BL: Bratislava, KI: Košice, NI: Nitra, PV: Prešov, TA: Trnava, TC: Trenčín ZI: Žilina.

Source: own elaboration (SPSS software output).

The given correspondence table represents a standard cross table that enables a sorting of procedure number according to fields and regions, while presenting row and column marginal totals/sums (Active Margin). These data represent the output for calculating the row profiles and column profiles in the following tables. We expect from the CA that the mutual category relations of a row variable *“Region”* and a column variable *“Field”* will enable the analysis more complexly within one correspondence map. The correspondence table contains the absolute numbers that are classified according to fields and regions. In the first row of the row profile table – in Bratislava Region, Otorhinolaryngology has a value of 0.564. It is a share of row total for Bratislava Region and a number of procedures in Otorhinolaryngology (1090/615). Otorhinolaryngology in the Bratislava Region has a value of 0.084 (7290/615) in the table of column profiles. In Tables 3 and 4, we present the given weights (mass) for a combination of row and column categories of variables *“Region”* and *“Field”* that form a basis for visualization of three graphs: graph of row points for *“Region”*, graph of column points for *“Field”* and Correspondence map in which both points are visualized.

**Table 3:** **Masses of row profiles**

**Region**	**Field**					
	**Surgery**	**Gyn**	**Ophthal**	**ORL**	**Urology**	**Active margin**
BL	.416	.000	.003	.564	.017	1.000
TA	.250	.009	.000	.524	.218	1.000
TC	.244	.019	.005	.680	.052	1.000
NI	.102	.008	.008	.715	.167	1.000
ZI	.245	.008	.024	.663	.060	1.000
BC	.667	.101	.011	.211	.009	1.000
PV	.113	.013	.019	.838	.017	1.000
KI	.099	.034	.018	.561	.288	1.000
Mass	.214	.021	.015	.634	.117	

Source: own elaboration (SPSS software output).

**Table 4:** **Masses of column profiles**

**Region**	**Field**					
	**Surgery**	**Gyn**	**Ophthal**	**ORL**	**Urology**	**Mass**
BL	.184	.000	.018	.084	.014	.095
TA	.070	.025	.000	.049	.111	.060
TC	.078	.063	.024	.074	.030	.069
NI	.042	.033	.048	.100	.126	.088
ZI	.224	.071	.321	.205	.101	.195
BC	.190	.296	.048	.020	.004	.061
PV	.103	.125	.250	.258	.028	.195
KI	.109	.388	.292	.210	.584	.237
Active margin	1.000	1.000	1.000	1.000	1.000	

Source: own elaboration (SPSS software output).

Masses for individual combinations of categories of two variables *“Region”* and *“Field”* in the table of row profiles will be transferred into the correspondence graph as a point. These weights represent weighted frequencies – numbers of each row point. “Total Mass”– a weight represents an average row profile. In this stage of the analysis, we may observe that in Bratislava Region, Otorhinolaryngology and Surgery have the largest row mass, in Trnava, Trenčín, Nitra, Žilina and Košice Regions dominates Otorhinolaryngology, and only in Banská Bystrica Region dominates Surgery.

In the table of column profiles, the item Mass represents an average column profile. The largest share of surgical procedures is recorded for Žilina Region; in Gynecology, Košice Region; in Ophthalmology, Žilina Region; in Otorhinolaryngology, Prešov Region and in Urology, an extremely high share is connected to Košice Region of the whole Slovak area. The CA compares the individual row and column profiles with average row and column profiles by contrast to our descriptive analysis of row and column profile. In the summarizing table (Table 5), there is given the data of contingency table approximation by means of its decomposition into singular values. This table is the most important table in the correspondence analysis.

**Table 5:** **Singular values of analyzed dimensions**

**Summary**								
**Dimension**	**Singular value**	**Inertia**	**Chi Square**	**Sig.**	**Proportion of Inertia**		**Confidence singular value**	
					**Accounted for**	**Cumulative**	**Standard deviation**	**Correlation**
								**2**
1	.406	.165			.594	.594	.008	.290
2	.310	.096			.346	.940	.008	
3	.118	.014			.050	.990		
4	.053	.003			.010	1.000		
Total		.278	3197.115	.000^a^	1.000	1.000		

^a^28 degrees of freedom.

Source: own elaboration (SPSS software output).

In the Table 5, there are presented four dimensions, as well as singular values that are assigned to them. Singular value is a root of its own number and it is interpreted as a maximum value of canonical correlation among the categories of variables in the analysis of a given dimension/measure. Reliability of singular values may be determined by its standard deviation. The first and the second dimension have the same standard deviation (SD = 0.008). It is a condition of using the correspondence map. Correlation of 0.290 indicates a closeness of dependence of the first two dimensions. Cohen [33] claims that it is in the superior limit of a small correlation. Correlation presented in the summarizing CA table has larger value (0.290) as Cramer’s V (0.264). The CA uses chi-square statistics to calculate a significance of total inertia – dispersion. In our case, Pearson chi-square value is equal to 3197.115 and p value (Sig) is 0.000. We may find the same value in the classic chi-square test of independence. CA belongs to the group of methods called “Data reduction” [27]. In our case, we extract four dimensions and our primary aim is to obtain as the largest share of explained variable as possible using as the smallest number of dimensions as possible. In two columns of “Proportion of Inertia”, there is a share of explained variable for each dimension and in the second column there are cumulative shares of variability. The first dimension represents 59.4% of variability and the second dimension represents only 34.6% of variability. Together, they represent 94% of dispersion and this is very positive. The third dimension represents only 5% of variability and the fourth dimension represents only 1% of variability. It means that there is a gap between the second and third dimension in a share of explained variability and the contribution of the last two dimensions (the third and the fourth) to totally explained variability is negligible. We lost only 6% by projection of five-dimensional pivot table to two-dimensional. We are sure that the relations and details within an original five-dimensional structure will be transferred to two dimensions in a correspondence map with a sufficient accuracy. It is very positive when two dimensions cover more than 90% of dispersion. If total explained share is smaller than 50%, then the correspondence analysis is not a suitable choice. Profile visualization is one of the advantages of correspondence analysis, because it properly enables to identify proximity or distance between row and column points, similarity, or profile differences in the correspondence map. We can analyze what is listed in the row point graph or column point graph as the dispersions in the row and column profiles are the same. This is considered as a curiosity of correspondence analysis. Therefore, some analysts use only a correspondence map without any graphs of row and column points.

**Figure 2:**
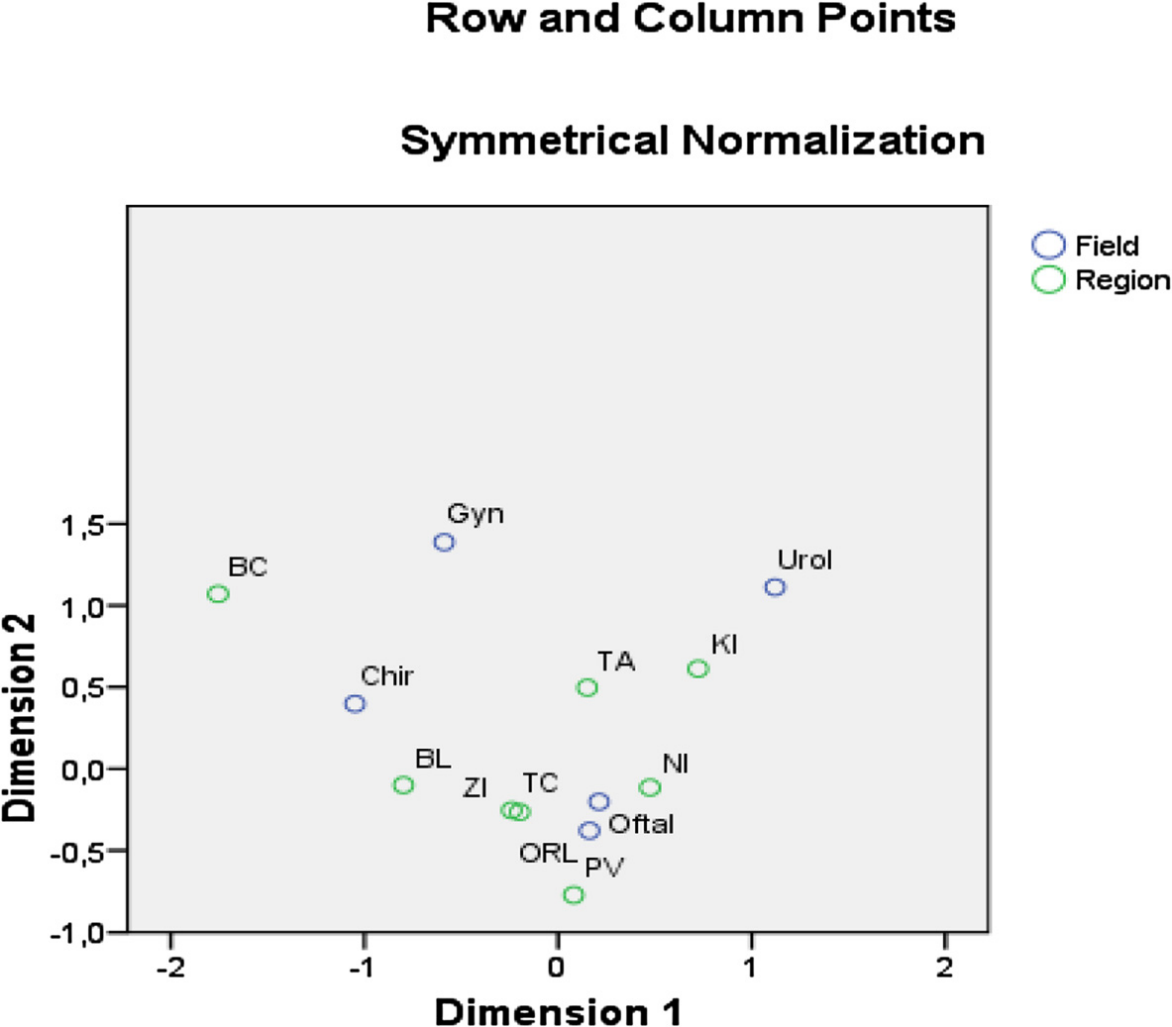
**Correspondence map (bi-plot) of regions and fields of non-hospitalized juniors.**

The correspondence map (Figure 2) is a combined graph of column and row profiles. Closeness or distance of these points may be perceived as a similarity that reflects association of categories of row and column variable that are projected in a form of points into two-dimensional Euclidean space of correspondence map. In the correspondence map, there are illustrated regions and fields from a point of view of a share (structure) of non-hospitalized juniors.

- Trenčín and Žilina Regions have the closest profile models, as well.
- We may identify a cluster of four regions (Trenčín, Žilina, Nitra and Prešov) that are associated with two fields (Ophthalmology and Otorhinolaryngology). In the row profile, these regions have the largest masses, or weighted frequencies of non-hospitalized juniors:
  - In Otorhinolaryngology: Trenčín Region = 0.680, Žilina Region = 0.663, Prešov Region = 0.838, Nitra Region = 0.715.
- Gynecology is the farthest field from this cluster together with Urology. Row and column profiles indicate the least similar in relation to the regions (of a given cluster). Outside that cluster, we observe the similarity between column profile of Gynecology and row profile of Košice Region, but the association will be smaller.
- Surgery has almost similar profile as Bratislava Region, and we suppose a certain small level of association here.

We may state the explicit conclusions, while dealing with interpretation of points’ similarity of row and column variables within that cluster. Interpretation of similarity outside the cluster is more subjective. There are no available rules, therefore we can subjectively conclude to a small similarity or its substitution with dissimilarity.

The development of number and structure of the DS procedures in juniors in the individual regions that was found out by the CA analysis offered us valuable information necessary for projection of specialized clinics of DS of a specific types of procedures, to support the formation of new ones (also potential state support), for politics of price making (in available data of the DS procedure risk level), system bed reduction and also reduction of insufficiently used bed units in the hospitals to optimize the control processes in the health system. Specialization of the clinics to the clinics of the DS is highly complex issue and it requires many system solutions. One method will not solve this issue. It is difficult to define clear statements and specialized fields, despite the fact that the CA offered us many valuable information of regions’ similarity and associations of specialized fields in them. You can enter many other significant factors into the process of the clinics’ specialization: clinics’ and health companies’ ownership, number and type of specialized field of the DS in a clinic, demographic aspects of inhabitants in a specific region, commorbity, availability of health care, performances’ risk of the DS, regional disparities, etc. These components in the health system require subsequent process of the analyses and aggregations of the outputs from them. The CA outputs offer us many possibilities in analyzing a group of similar and different regions and in choosing of suitable complementary and analytical methods. One of them is also a structural analysis via MIC-MAC method [34]. We may achieve a wide spectrum of point of views and expert evaluations that may help in setting effective specializations of the DS clinics and subsequently in its development as a system via aggregating the results of numerous methods.

## Discussion

The similarity of analyzed profiles, as an output of the CA, determines characteristic settings of components of the DS system in the individual regions. This similarity or disparity is inevitable to closely analyze by other methods, while considering many particularities, as it was already mentioned. The CA also determines the analytical direction, except these outputs – a choice of other methods and components of the DS system that are necessary to monitor and analyze. We observe in this fact further contribution of the CA application.

In our analysis, we abstracted from information about the type of clinics the DS procedure was realized (state vs. private). This factor is very important in the process of the DS development as the activities of some private clinics (also newly-created) lead to intentional and non-regulated by state transfer of inquiry. The primary aim is to get a financial profit for HCP (the provided shorter waiting time, on the expense of higher, or total financial payments by patient for procedure). Workplace motivation, indication experience and operational skills of a surgeon, qualification, technical and pharmacological possibilities of anesthesiologist, quality of home nursing and patient’s motivation are important for the development of DS without any regard to procedure realized by classic hospital, newly-created specialized clinic or out-patients clinic. However, the reality is different. Besides competition among DS workplaces, regulation of competition is also inevitable among the DS workplaces and standard bed departments of hospitals. At present, more than half of all surgical procedures are possible to realize in the DS mode, while augmentation of procedure number suitable to DS does not indicate any threat for hospitals, as there are wide spectrum of a difficult operation that will always belong to standard bed facility. It is connected with a level of patients’ risk that is influenced by co - morbidities. These patients have to be admitted to bed facility immediately after realization of a simple operational procedure in order to make available a surgical healthcare for them in the intensive care units. Also, the complications after DS procedure are possible to solve only in a standard bed facility with all corresponding background. Therefore, it is inevitable to provide a simultaneous influence among the departments – DS, as well as the standard bed ones. In this aspect, there is important a formation of such conditions that will provide a transparent competition among HCP. It would be also appropriate specialize the DS clinics and hospitals in chosen types of procedures in the individual specialized fields regarding the regional differences in number and structure of realized DS procedures. It is a traditional process in the clinics in Europe and the USA, where the centre of DS were established and they have been functioning for many decades. They are based on very close specialization of procedures. In the process of bed and department reduction, some small hospitals should transform into the DS units. This process will decrease the costs and they will provide almost the same variability of operational procedures except of complicated, and rarely realized. A patient who suffers from a serious illness should be treated in some of the hospitals, or in other specialized facility, where a top healthcare would be provided to him as such workplace will be specialized in a given type of operation with rich experiences, including technical background for subsequent treatment. Many private clinics in Slovakia also use their own methodologies of data reporting of surgical procedures. They are deformed by present influence of HIC price strategies. As a consequence of aforementioned, many hospitals intentionally transform their procedures from DS procedure groups into a group of so-called “3-day” procedures (referred to as “Separately reimbursed procedures”), while a patient will be firstly diagnosed as risky without any complementary analytical basis, or they realize all the procedures exclusively by traditional form of hospitalization and they apply their own policy of financing and healthcare organization. System of HIC reimbursement is set in a way that if a given type of procedure, which is specified for realization by DS form is present in the hospitals and the hospital realizes it by traditional form of hospitalization without any adequate reasons, the HIC will reimburse only for a procedure that is only equal to a procedure realized by the DS form. These factors – competition among DS, own system of the DS procedure reporting, intentional transfer of procedures from various payment categories (one-day, 3-day or hospitalized) represent deep system defects that are inevitable to correct and eliminate legislatively as they will always prevent the DS development.

Individual position in this system has also a price policy of the HIC that is predominantly characteristic of different approach to pricing in the DS procedures. The HIC set different prices for the DS procedures that are differentiated from the HCP, while their preferential criteria are not clear. There absents a standardization of the DS procedures, which prevents price comparison among them in Slovakia. On the other hand, especially private clinics refuse to provide the necessary data to the HIC that are also inevitable for price setting of the individual procedures. If we consider a fact that present DS procedure prices are formed without any calculations of the individual items and the resulting prices for HCP are usually a result of lobbing, the pricing of the DS procedures will be a cardinal issue and a barrier in further DS development. It is difficult to estimate an impact of system unitarisation of public health insurance in this case (at present, it is stopped by government), even we may expect a certain level of consistency and simplification of the whole process. If we observed experiences of other countries, especially in those where strong integration efforts of patients’ treatment prevail, there was a significant development of DS. Here belong for instance health systems with competitive HCP, such as in the USA [22], or state-controlled health systems, such as in Australia and the UK [10]. The given issues of price making of the DS procedures will not solve any planned and classified system of Diagnoses Related Groups (DRG) that is not implemented in our country as the only country of the EU countries. The German planned DRG system does not contain the DS procedures, thus they must be solved separately. This system is planned to be implemented in our country in 2015.

It is inevitable to determine a definite framework and rules for all subjects of the DS system for its development and proposed specialization of the DS clinics. It is an exact definition of patients who are suitable for treatment in the DS mode with a high emphasis on the DS workplace outside the hospital facility [35-37]. Consequently, there is a necessity of analyses realization that will focus on revelation of risk groups of patients in order to correctly localize a realization of the given procedure type [38]. This is connected with a determination of clearly defined requirements of personal and technical equipment of given workplaces. In realizing the DS, we do not have a high-class system of subsequent healthcare, therefore the DS is not suitable for patients who live alone in Slovakia (it is related to older patients). Therefore, the government support is necessary in supporting the institutions of the type of agencies as home nursing, or after-treatment geriatrics centres. At present, the expansion of DS is prevented by geographical conditions and social situation in Slovakia. The presented aspects of the DS development have a systemic character and they are necessary to be involved into strategic framework of the Slovak health system. This will probably support the DS development in Slovakia that may be comparable with foreign countries.

## Conclusions

In the last two decades, there was a reduction of beds as well as an average length of stay in a hospital in the health systems of most of the European countries [10]. Intensity of this process was not the same, which is also declared by significant differences among individual countries from the point of view of the total amount of hospital sources and activities, average length of patient’s stay in a hospital, shares of realized procedures by the DS form on total procedures, etc. Solution of this issue of the DS efficiency in the health system is very difficult. This is connected with conceptual and methodological issues of its measurements. These issues were advised also by international institutions, such as Eurostat, OECD and WHO, which have been gathering data of surgical procedures as a part of total data collection of healthcare for many years. Despite this fact, there absents a single international classification of procedures that would enable a comparison of countries in their DS use and also support determinations of comparable platform. It is very complex issue that is necessary to be solved at the micro-level (in the health systems of those countries). In rationally financed systems of healthcare, it is obvious that the DS is less expensive and its savings are used for financing more difficult surgery, which focuses on highly-difficult bed centres that gather professional teams and technologies. We focused on the analysis of the DS status in Slovakia in this article via CA application. Results of CA tell us about the similarity of specialized departments and regions in the day surgery system. It is not possible to secure, technically or financially, functioning of day surgery in many specialized fields in the hospital, but it is inevitable to specialize according to patient morbidity, catchment area, technical and economic possibilities of hospital and subsequent healthcare system in specific areas. Results of CA help to make the process of creating specialized health care centres to improve the quality and efficiency, so to avoid unnecessary duplication of services and ensure better use of scarce labour resources. Since it is not possible to implement data of seniors for CA, in the case of elective specialty clinics is important to use appropriate analytical tools whose output would reflect morbidity rate of patients - seniors and their comorbidities [39].

The CA method provided us much information of regions’ similarities from the point of view of using the DS as well as associations of the specialized fields in them. Its outputs form a platform in choosing the related analytical methods and constructions of complex system tools in a process of the DS development in Slovakia. Slovakia shows a minimum rate of these procedures for 15 years of the DS existence there. DS availability is especially dictated by HIC via concluding (non-concluding) the contractual relation with the HCP, economically verified pricing as well as price differentiation for individual types of HCP. This caused an implantation of unfair practices of some providers that were related to transfer of patients into different DS modes, as well as manipulation of reported procedures, inquiry exclusion, etc. Intensive process of DS development requires effective legislative support with some restrictive elements that would increase an intensification of healthcare process. Other issues is also important – the issue of state vs. private ownership of HCP, as well as state vs. private ownership of HIC. The DS system will remain fractured without finding a consensus of more actors of healthcare system. It will also not be possible to proceed a rationalization and specialization of DS workplaces, bed optimizing and bed departments as there will absent motivation of doctors and patients as well as there will prevail tendency efforts of many HCP to prefer the traditional hospitalization. This trajectory will have a negative impact on the DS system and also efficiency of the whole health system in Slovakia due to its cyclic linkage of causes and consequences.

### Endnotes

^a^Countries, which reached a large overvaluation of procedures number due to influence of own applied methodology of counting and reporting the surgical procedures are as follows: Belgium, Germany and Slovenia, a medium overvaluation: Estonia, Italy, Luxembourg, the Netherlands, Switzerland and the United Kingdom.

^b^Regulation of Ministry of Health of the Slovak Republic of day surgery procedures No. 06937/2006 from 30.1.2006, Regulation of Ministry of Health of the Slovak Republic No.12225/2009 - of day surgery procedures and Regulation of Ministry of Health of the Slovak Republic No. 08465/2010.
